# Association between dietary vitamin intake and all-cause mortality in ovarian cancer patients: a prospective cohort study

**DOI:** 10.3389/fnut.2025.1554253

**Published:** 2025-05-30

**Authors:** Qin Ling, Linxiang Wu, Huishan Xu, Yang Zhang, Yuying Chen, Tingting Sun, Zuwei Zhang, Huiling Lai, Shasha He, Shuzhong Yao, Weipeng He, Guofen Yang

**Affiliations:** Department of Gynecology, The First Affiliated Hospital of Sun Yat-sen University, Guangzhou, Guangdong, China

**Keywords:** ovarian cancer, dietary vitamin intake, Cox regression, National Health and Nutrition Examination Survey, NHANES, all-cause mortality, survival

## Abstract

**Background:**

While various lifestyle factors have been implicated in cancer prognosis, the role of dietary vitamins in ovarian cancer survival is not well understood. This study aimed to investigate the association between dietary vitamin intake and all-cause mortality in ovarian cancer patients, presenting a potential modifiable avenue for improving outcomes.

**Methods:**

Data were obtained from 7 consecutive National Health and Nutrition Examination Survey (NHANES) cycles between 2003 and 2016, including 108 ovarian cancer patients. Mortality outcomes were ascertained by matching the National Death Index (NDI). To investigate the association between vitamin consumption and all-cause mortality, multivariate Cox proportional hazards models was employed. Non-linear relationships were further assessed through restricted cubic spline analyses and subgroup analyses were conducted to explore the influence of potential confounders.

**Results:**

A total of 108 ovarian cancer patients were included, of which 24 participants have died. In the adjusted model, higher vitamin A intake was significantly associated with an increased all-cause mortality (HR = 3.826; 95% CI = 1.378–10.627; *p* = 0.01). In contrast, higher intake of vitamins B1 and B2 was associated with improved survival (HR = 0.234, 95% CI = 0.077–0.71, *p* = 0.01; HR = 0.14, 95% CI = 0.048–0.408, *p* < 0.001). Vitamin C intake showed a complex relationship with survival: the highest tertile had an increased risk of death (HR_T3 vs. T1_ = 4.106; 95% CI = 1.294–13.032; *p* = 0.017), while the moderate tertile were just the opposite (HR_T2 vs. T1_ = 0.097; 95% CI = 0.013–0.75; *p* = 0.025). Non-linear associations were exhibited between vitamins A, B1, and B2 with all-cause mortality (*p*-non-linear = 0.007, 0.008, 0.027). Subgroup analyses revealed that the education level, racial and smoking status differences may cause difference in results.

**Conclusion:**

This study suggests that higher dietary intake of vitamin A may increase mortality risk in ovarian cancer patients, while vitamins B1 and B2 may offer potential survival benefits. The relationship between vitamin C and survival varied with intake levels. These results highlight the potential for personalized dietary interventions in ovarian cancer management.

## Introduction

Ovarian cancer remains a significant health challenge, with the highest mortality rate among gynecologic cancers globally. In 2020, it accounted for 3.7% of cancer cases and 4.7% of cancer deaths, ranking eighth in incidence among women (1).

The prognosis of ovarian cancer is heavily influenced by various factors, including cancer stage, histological grade, health status of patients, treatment approaches and so on (2). However, many of these risk factors are beyond control, highlighting the importance of identifying modifiable factors that impact patient outcomes. As a factor that can be easily controlled and adjusted, dietary factors offer a promising and controllable avenue for potentially enhancing the prognosis of cancer.

It is known that dietary vitamins are closely related to many physiological processes, such as immune function, cell repair and antioxidant (3), which provided relevant evidence for their association with the risk and prognosis of tumor. There are many studies have shown that dietary vitamins are associated with the prognosis of tumors. For example, Christine et al. found that antioxidant vitamin supplements, such as vitamins A, C, and E, were associated with increased breast cancer recurrence, while for non-antioxidant vitamins, such as vitamin B12, were linked to poorer disease-free and overall survival (4). However, the relationship between dietary vitamins and ovarian cancer prognosis remains inconclusive. In 2010, Cook et al. (5) reviewed the association between Vitamin D and ovarian cancer risk or mortality, but despite analyzing a significant amount of literature, they could not establish a clear link. In addition, other studies have yielded inconsistent findings. For instance, Sun et al. (6) found that higher pre-diagnosis intakes of vitamin C (HR = 0.43, 95% CI = 0.25–0.75, *p* < 0.05) and *β*-carotene (HR = 0.52, 95% CI = 0.31–0.87, *p* < 0.05) were associated with improved ovarian cancer survival. In contrast, a prospective cohort study by Gu et al. (7) on Chinese women found no evidence linking pre-diagnosis dietary supplement intake to ovarian cancer survival. These discrepancies underscore the need for further research to clarify the role of dietary vitamins in ovarian cancer prognosis.

This study aims to explore the relationship between dietary vitamin intake and all-cause mortality in ovarian cancer patients, utilizing data from the National Health and Nutrition Examination Survey (NHANES) to better understand how vitamin levels may affect survival outcomes. Ultimately, these findings could contribute to personalized dietary recommendations for ovarian cancer patients, offering a potential strategy to improve patient management and survival.

## Materials and methods

### Data source and study subjects

NHANES is a comprehensive, continuous survey designed to assess the health and nutritional status of the non-institutionalized civilian population in the United States (8). Conducted biennially by the National Center for Health Statistics (NCHS) of the Centers for Disease Control and Prevention (CDC), NHANES employs a complex multistage stratified sampling design to ensure representative data collection. The survey combines interviews, physical examinations, and laboratory tests administered by trained health professionals at participants’ homes and mobile examination centers. This multifaceted approach yields valuable data on health topics such as dietary habits, chronic conditions, and risk factors, significantly contributing to public health research and policy. For further details and dataset access, please visit the official NHANES website^1^.

**Figure 1:**
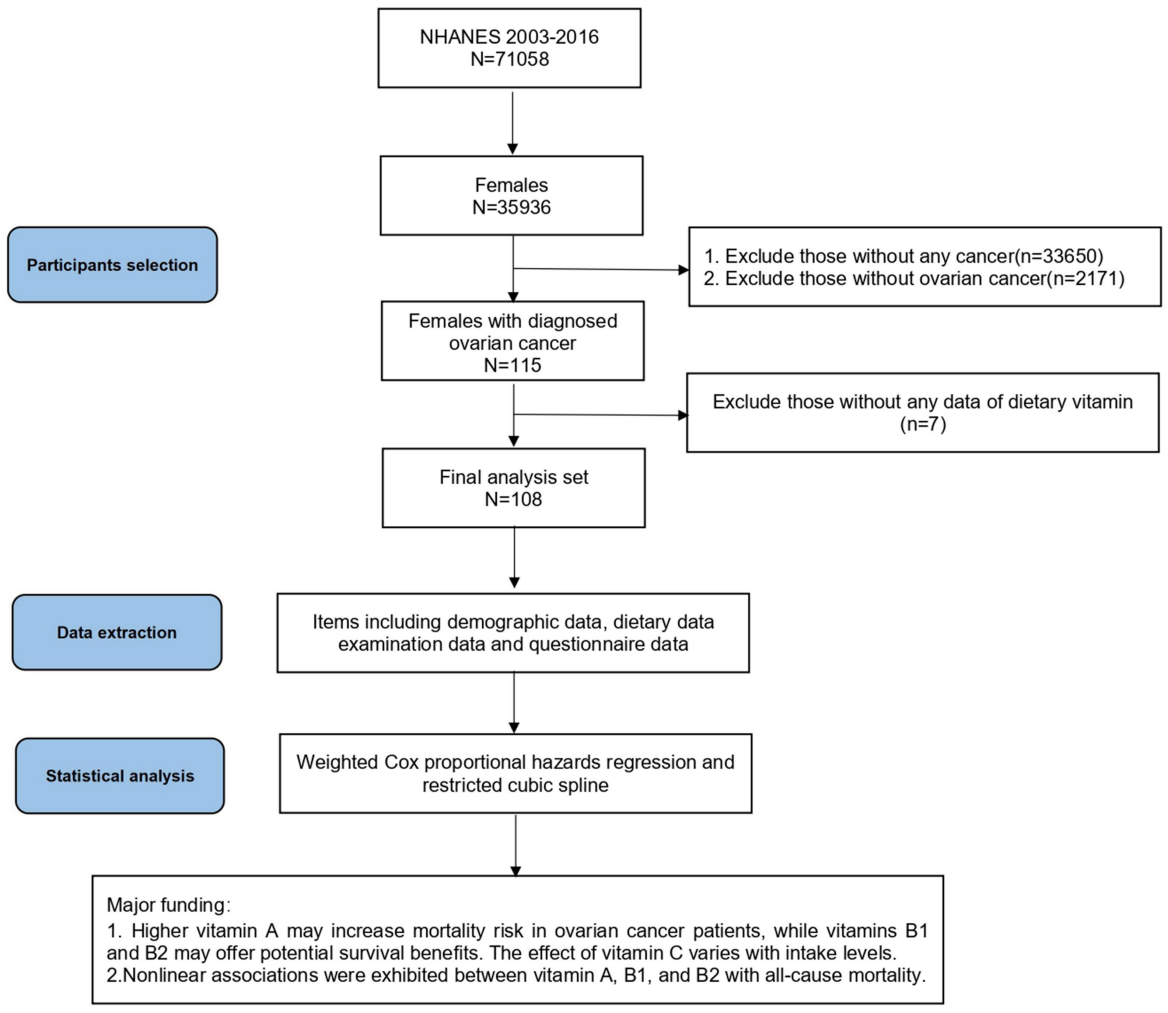
Flow chart and major findings of this study from the NHANES 2003–2016. NHANES, National Health and Nutrition Examination Survey.

Participants in this study were ovarian cancer patients with complete dietary and health data, and were identified as women diagnosed with ovarian cancer based on their responses to the Medical Status Questionnaire (MCQ). The participant selection process for this prospective cohort study is illustrated in Figure 1. We analyzed data from 2003 to 2016, encompassing 71,058 participants. Inclusion criteria were: (1) female participants; (2) patients with ovarian cancer; (3) NHANES participants with dietary data. Among of the 35,936 female participants in the NHANES 2003–2016, we excluded 33,650 patients without any cancer and 2,171 patients with other cancers than ovarian cancer. After excluding 7 people with missing dietary vitamin data, 108 participants were included in the final analysis.

### Definition of ovarian cancer

In this study, only female participants diagnosed with ovarian cancer were included. Women were considered eligible if they responded “yes” to the MCQ item: “Ever told you had cancer or malignancy?”. Participants specifying “Ovary (ovarian)” in response to the subsequent question about cancer type were classified as ovarian cancer patients.

### Assessment of dietary vitamin

Dietary intake of vitamins was assessed using a 24-h dietary recall questionnaire. Participants from the biennial cohorts (2003–2016) completed two dietary recalls. The first interview (DR1TOT) was conducted in person at the Mobile Examination Center (MEC), while the second (DR2TOT) was conducted by telephone 3 to 10 days later.

For analysis, we considered the following vitamins: A, B1, B2, B12, C, D, and K. Vitamin D was excluded from this study due to missing values among the 108 participants, which could compromise sample size and accuracy. Dietary intake estimates were derived from the average of the two dietary recalls, or a single recall if the second was unavailable.

### Outcome ascertainment

The outcome of interest was all-cause mortality. The mortality data for participants in NHANES were obtained through a comprehensive probabilistic linkage with the National Death Index (NDI)^2^. This linkage process, detailed in prior studies, has demonstrated reliable death ascertainment. Each NHANES participant’s mortality status was confirmed by matching their records with death certificate information from the NDI. Additionally, vital status was verified through linkages with the U.S. Social Security Administration and active follow-ups with survey participants. The follow-up period extended from the baseline examination date (2003–2016) until either the date of death was recorded or the study concluded on December 31, 2019.

### Covariates

Covariates were chosen based on their biological plausibility and established associations with ovarian cancer or dietary vitamins (9). We comprehensively analyzed the baseline characteristics, examination data, and related questionnaire responses of the included subjects.

The demographic variable was gathered from NHANES household interviews, including age; ethnicity/race (Mexican American, other Hispanic, non-Hispanic white, non-Hispanic black, other race); socioeconomic status as measured by poverty–income ratio (PIR), a ratio of a family’s income to their poverty (low: <1.30, moderate: 1.30–3.50; high: ≥3.50); education level (less than 9th grade, 9-11th grade, 12th grade or above); marital status (married, widowed/divorced/separated, and never married).

Physical examination included measuring each participant’s height and weight and body mass index (BMI) was calculated as weight divided by height squared (kg/m^2^).

The smoking status of individuals was recorded as never smoking (smoked fewer than 100 cigarettes in life), former smoking (smoked more than 100 cigarettes in life but currently not smoking), and current smoking (smoked more than 100 cigarettes in life and currently smoking). Current drinking was defined as having consumed at least 12 alcoholic drinks per year. According to the recommended weekly amount of moderate-vigorous activity, physical activity was categorized as below or exceeded the guidelines.

Additionally, we considered factors such as age at cancer diagnosis, the presence of multiple cancer types, and the use of female hormones, as these may influence outcomes. Further data analysis was conducted on these variables.

### Statistical analysis

Descriptive statistics were computed for general and dietary characteristics. Continuous variables were reported as means with standard deviations (SD), while categorical variables were presented as frequencies and percentages. To account for the NHANES sampling design, all analyses included sample weighting, strata, and primary sampling units, following CDC recommendations (10). The sample weight was adjusted by dividing by 7 for the seven continuous cycles.

We plotted receiver operating characteristic (ROC) curves to assess the relationship between dietary vitamin intake and ovarian cancer survival. The maximum value of the Youden index was identified as the cut-off point for tertile 1 and tertile 2. After removing the value of tertile 1, the above steps were repeated to obtain the cut-off value for tertile 2 and tertile 3. Finally, dietary vitamin intake was categorized into three tertiles (11).

Cox proportional hazards regression models were employed to calculate adjusted hazard ratios and 95% confidence intervals for the association between dietary vitamin intake and all-cause mortality in ovarian cancer patients. Univariate models were initially used to explore covariates related to survival, followed by multivariate models that adjusted for statistically significant covariates identified in the univariate analysis. The lowest tertiles of vitamin intake served as the reference group, with fully adjusted models yielding the final results.

Kaplan–Meier curves showed crude survival probabilities for each vitamin, which generated to verify consistency with Cox regression findings. A nomogram incorporating statistically significant variables from the Cox models was developed to visualize our results.

Multicollinearity among variables was assessed using the Variance Inflation Factor (VIF), with values exceeding 10 indicating serious multicollinearity (12). We utilized restricted cubic splines (RCS) to examine non-linear associations between dietary vitamins and ovarian cancer survival, treating vitamin intakes as continuous variables.

Subgroup analyses compared the tertile 1 and tertile 2 to the lowest tertile of dietary vitamins based on all the possible confounders.

All the above statistical analysis was performed by R software (version 4.4.1; https://www.r-project.org) and SPSS (version 25). A two-tailed *p* < 0.05 was considered statistically significant.

## Results

### Baseline characteristics of the study participants

Our final analysis included 108 patients diagnosed with ovarian cancer, among whom 24 had died. The baseline characteristics of these participants are detailed in Table 1. The mean age of the population was 60.15 years (SD 15.35), with a mean age at diagnosis of 43.29 years (SD 16.22). The average BMI was 30.73 kg/m^2^ (SD 7.64). The demographic breakdown included 18.5% Mexican American, 9.3% other Hispanic, 45.4% non-Hispanic white, 21.3% non-Hispanic black, and 5.6% other races. Regarding education, 33.3% had less than a 9th-grade education, while 40.7% had attained higher education. Economic analysis revealed that 39.8% of patients had a household income below 1.30, indicating a relatively low income.

**Table 1:** Baseline characteristics of 108 OC patients in the NHANES 2003–2016.

Characteristics	All participants
Age, years	60.15(15.35)
Age at diagnosis, years	43.29(16.22)
BMI, kg/m^2^	30.73(7.64)
Race/ethnicity, *n*(%)	
Mexican American	20(18.5%)
Other Hispanic	10(9.3%)
Non-Hispanic white	49(45.4%)
Non-Hispanic black	23(21.3%)
Other race	6(5.6%)
Educational level, *n*(%)	
Less than 9th grade	36(33.3%)
9–11th grade	28(25.9%)
12th grade or above	44(40.7%)
PIR, *n*(%)	
<1.30	43(39.8%)
1.30–3.50	29(26.9%)
≥3.50	27(25.0%)
Smoking status, *n*(%)	
Never smoking	67(62.0%)
Former smoking	14(13.0%)
Current smoking	27(25.0%)
Drinking statues, *n*(%)	
Current drinking	52(48.1%)
Non-current drinking	50(46.3%)
Physical activity, *n*(%)	
Below the guideline	49(45.4%)
Exceed the guidelines	42(38.9%)
Marital status, *n*(%)	
Married	48(44.4%)
Widowed/divorced/separated/never married	60(55.6%)
Use of female hormones, *n*(%)	
Yes	38(35.2%)
No	70(64.8%)
More than one type of cancer, *n*(%)	
Yes	35(32.4%)
No	70(64.8%)
Dietary vitamin intake	
Vitamin A, mcg	546.53(402.83)
Vitamin B1, mg	1.29(0.57)
Vitamin B2, mg	1.71(0.81)
Vitamin B6, mg	1.54(0.73)
Vitamin B12, mcg	3.94(2.86)
Vitamin C, mg	80.44(69.76)
Vitamin K, mcg	95.04(159.95)

Data were expressed as means (SD) for continuous variables, and frequency (percentage) for categorical variables. All estimates were weighted to be nationally representative.

BMI, body mass index; SD, standard deviation.

In terms of lifestyle factors, 62.0% of patients had never smoked, 25.0% were current smokers, and 48.1% were current drinkers. Furthermore, 45.4% of patients engaged in physical activity below guideline recommendations. Dietary intake analysis showed that the average vitamin A intake was 546.53 mcg (SD 402.83), and vitamin C intake was 80.44 mg (SD 69.76).

These factors may significantly impact patient outcomes. For example, participants with higher BMI and older age tended to have higher mortality rates, leading to an increase of the adverse outcome in the corresponding group, and thus affecting the accuracy of the results. Therefore, it is necessary to analyze the baseline characteristics of participants with different vitamin intake levels, which was shown in the subsequent study.

### Association between dietary vitamin intake and all-cause mortality

Univariate Cox regression analysis (Supplementary Table 1) indicated that age (*p* = 0.002) and smoking status (*p* = 0.028) were significant covariates. We included these factors in subsequent multivariate analyses, establishing three models. The crude model was unadjusted, while Model I adjusted for age, and Model II further included smoking status. We used Model II as our final conclusion.

Table 2 presents the associations between dietary vitamins and all-cause mortality. In the fully adjusted multivariate models, higher vitamin A intake was associated with increased all-cause mortality (HR_T3 vs. T1_ = 3.826; 95% CI = 1.378–10.627; *p* = 0.01), which was consistent with the results in the crude model (HR_T3 vs. T1_ = 3.564; 95% CI = 1.414–8.986; *p* = 0.002) and Model I (HR_T3 vs. T1_ = 2.802; 95% CI = 1.098–7.15; *p* = 0.031).

**Table 2:** Association between dietary vitamin intake and all-cause mortality in ovarian cancer patients, Nation Health and Nutrition Examination Survey 2003–2016.

Dietary vitamin intake	Tertiles	Crude Model HR (95%CI)	P	Model I HR (95%CI)	*P*	Model II HR (95%CI)	*P*
Vitamin A	T1(<592mcg)	Ref		Ref		Ref	
	T2(592–899mcg)	2.041 (0.664, 6.278)	0.02	1.273 (0.402, 4.033)	0.682	1.747 (0.534, 5.718)	0.356
	T3(>899mcg)	3.564 (1.414, 8.986)	0.002	2.802 (1.098, 7.15)	0.031	3.826 (1.378, 10.627)	0.01
Vitamin B1	T1(<0.82 mg)	Ref		Ref		Ref	
	T2(0.82–1.63 mg)	0.189 (0.632, 0.564)	0.189	0.234 (0.077, 0.71)	0.01	0.28 (0.09, 0.873)	0.028
	T3(>1.63 mg)	0.796 (0.322, 1.966)	0.13	0.79 (0.319, 1.958)	0.611	0.967 (0.368, 2.542)	0.946
Vitamin B2	T1(<0.90 mg)	Ref		Ref		Ref	
	T2(0.90–2.04 mg)	0.132 (0.045, 0.384)	0.055	0.14 (0.048, 0.408)	< 0.001	0.166 (0.053, 0.518)	0.002
	T3(>2.04 mg)	0.488 (0.183, 1.301)	0.302	0.456 (0.171, 1.218)	0.117	0.535 (0.193, 1.486)	0.23
Vitamin B6	T1(<1.24 mg)	Ref		Ref		Ref	
	T2(1.24–1.98 mg)	0.183 (0.052, 0.643)	0.244	0.274 (0.076, 0.993)	0.049	0.307 (0.084, 1.121)	0.074
	T3(>1.98 mg)	0.854 (0.352, 2.068)	0.558	0.986 (0.405, 2.401)	0.975	1.086 (0.436, 2.702)	0.86
Vitamin B12	T1(<3.83mcg)	Ref		Ref		Ref	
	T2(3.83–4.41mcg)	3.694 (1.372, 9.948)	0.008	2.951 (1.088, 8.009)	0.034	2.505 (0.895, 7.013)	0.08
	T3(>4.41mcg)	1.568 (0.604, 4.071)	0.233	1.458 (0.559, 3.802)	0.441	1.639 (0.59, 4.557)	0.343
Vitamin C	T1(<115.13 mg)	Ref		Ref		Ref	
	T2(115.13–141.25 mg)	0.122 (0.016, 0.912)	0.006	0.106 (0.014, 0.795)	0.029	0.097 (0.013, 0.75)	0.025
	T3(>141.25 mg)	2.571 (0.864, 7.643)	0.139	3.193 (1.063, 9.594)	0.039	4.106 (1.294, 13.032)	0.017
Vitamin K	T1(<115.13 mg)	Ref		Ref		Ref	
	T2(115.13–141.25 mg)	0(0, 0)	0.997	0(0, 0)	0.997	0(0, 0)	0.997
	T3(>141.25 mg)	0.364 (0.049, 2.704)	0.323	0.223 (0.03, 1.674)	0.145	0.252 (0.033, 1.911)	0.182

Crude Model: unadjusted any factor.

Model I: multi-factor model adjusted for age.

Model II: multi-factor model adjusted for Model I + smoking status.

HR: hazard ratio; 95% CI: 95% confidence interval; Ref: reference.

Additionally, the middle tertiles of vitamin B1 and vitamin B2 were associated with improved survival among ovarian cancer patients, showing statistical significance in both Model I (Vitamin B1: HR_T2 vs. T1_ = 0.234; 95% CI = 0.077–0.71; *p* = 0.01. Vitamin B2: HR_T2 vs. T1_ = 0.14; 95% CI = 0.048–0.408; *p* < 0.001) and Model II (Vitamin B1: HR_T2 vs. T1_ = 0.28; 95% CI = 0.09–0.873; *p* = 0.028. Vitamin B2: HR_T2 vs. T1_ = 0.166; 95% CI = 0.053–0.518; *p* = 0.002).

For vitamins B6 and vitamin B12, Model I suggested potential protective effects (Vitamin B6: HR_T2 vs. T1_ = 0.274; 95% CI = 0.076–0.993; *p* = 0.049. Vitamin B12: HR_T2 vs. T1_ = 2.951; 95% CI = 1.088–8.009; *p* = 0.034), though no associations were found in Model II. Vitamin C intake showed a complex relationship: compared to the lowest tertile, those in the highest tertile had an increased risk of death (HR_T3 vs. T1_ = 4.106; 95% CI = 1.294–13.032; *p* = 0.017), while the middle tertile was associated with improved survival (HR_T2 vs. T1_ = 0.097; 95% CI = 0.013–0.75; *p* = 0.025).

In summary, higher intake of dietary vitamin A was associated with an increased risk of ovarian cancer mortality, while vitamins B1 and vitamin B2 showed potential positive effects on survival. The impact of vitamin C on prognosis varied with intake level: The highest tertile of vitamin C is detrimental to ovarian cancer patients, while the intermediate dose may be protective. The associations for vitamins B6, B12, and K were not statistically significant, indicating the need for further investigation.

From the above results, we can easily see the necessity to adjust for confounding factors. For example, in the adjusted models, where we adjusted for age and smoking status, the association between vitamin B1 intake in tertile2 and increased mortality became statistically significant (Crude Model: *p* = 0.025 vs. Model II: *p* = 0.028).

Moreover, we calculated the VIF for each variable to assess multicollinearity among them. All VIF values below 10, excluding the potential effect of collinearity between variables on the results (Supplementary Table 2). Kaplan–Meier curves (Supplementary Figure 1) aligned with our analyses, further validating our findings.

**Figure 2:**
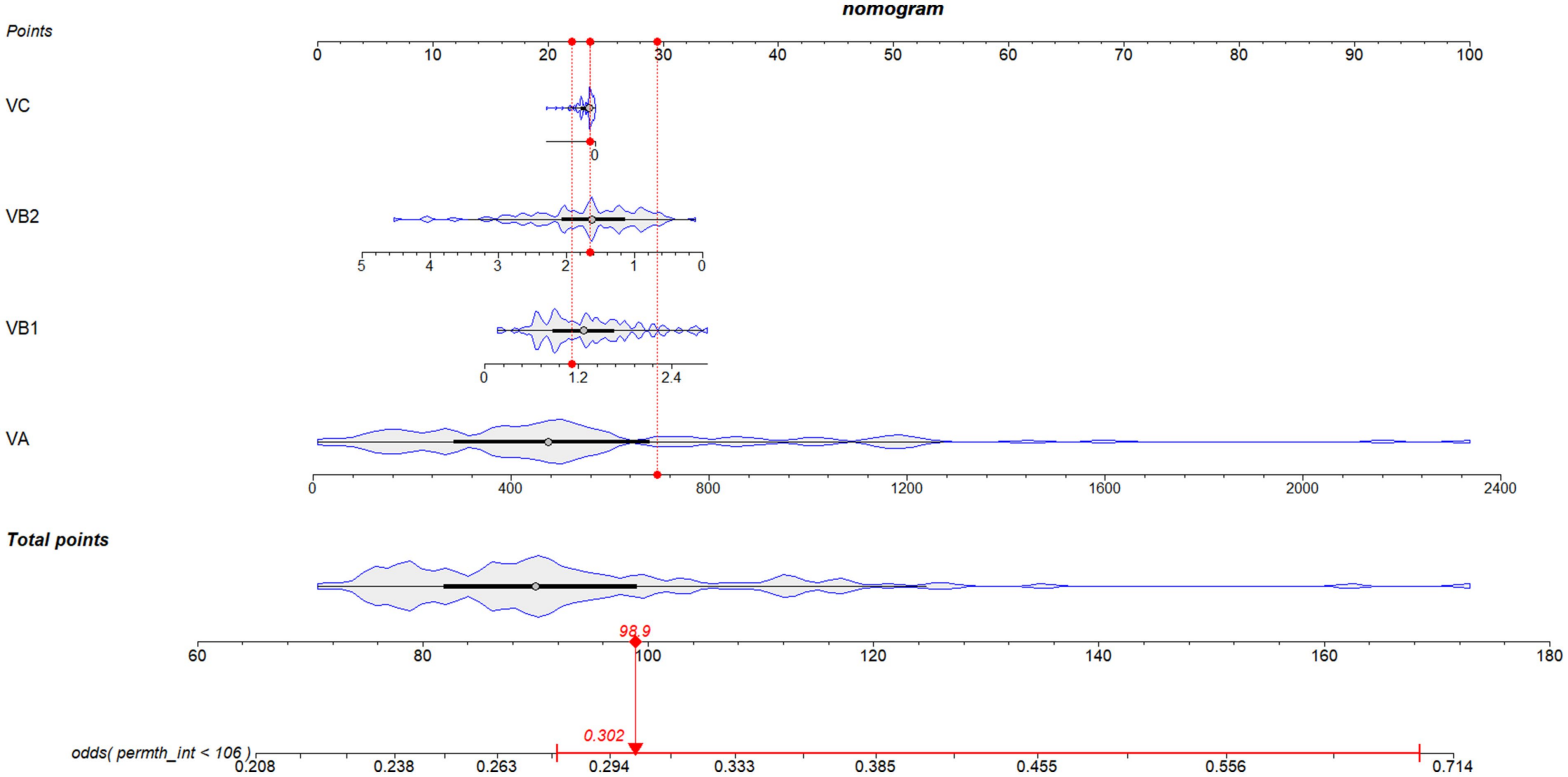
Novel diagnostic nomogram to predict the survival probability of ovarian cancer patients with different dietary vitamin intake levels. Each predictive risk factor is given a score according to its predictive value on the point scale axis. Scores for each single variable are added together to calculate the total points. Then the total points are converted into an estimate of the survival probability by projecting the total points to the lower probability axis.

To make our results more readable, we developed a nomogram incorporating statistically significant variables from the multivariate logistic regression analysis (Figure 2). Derived from the relationship between vitamin intake and survival outcomes, the nomogram allows healthcare providers to personalize treatment strategies and adjust dietary recommendations for ovarian cancer patients. This visual tool simplifies the interpretation of complex regression outcomes, facilitating evaluations and recommendations for ovarian cancer patients.

Curve-fitting association of dietary vitamin intake with all-cause mortality.

**Figure 3:**
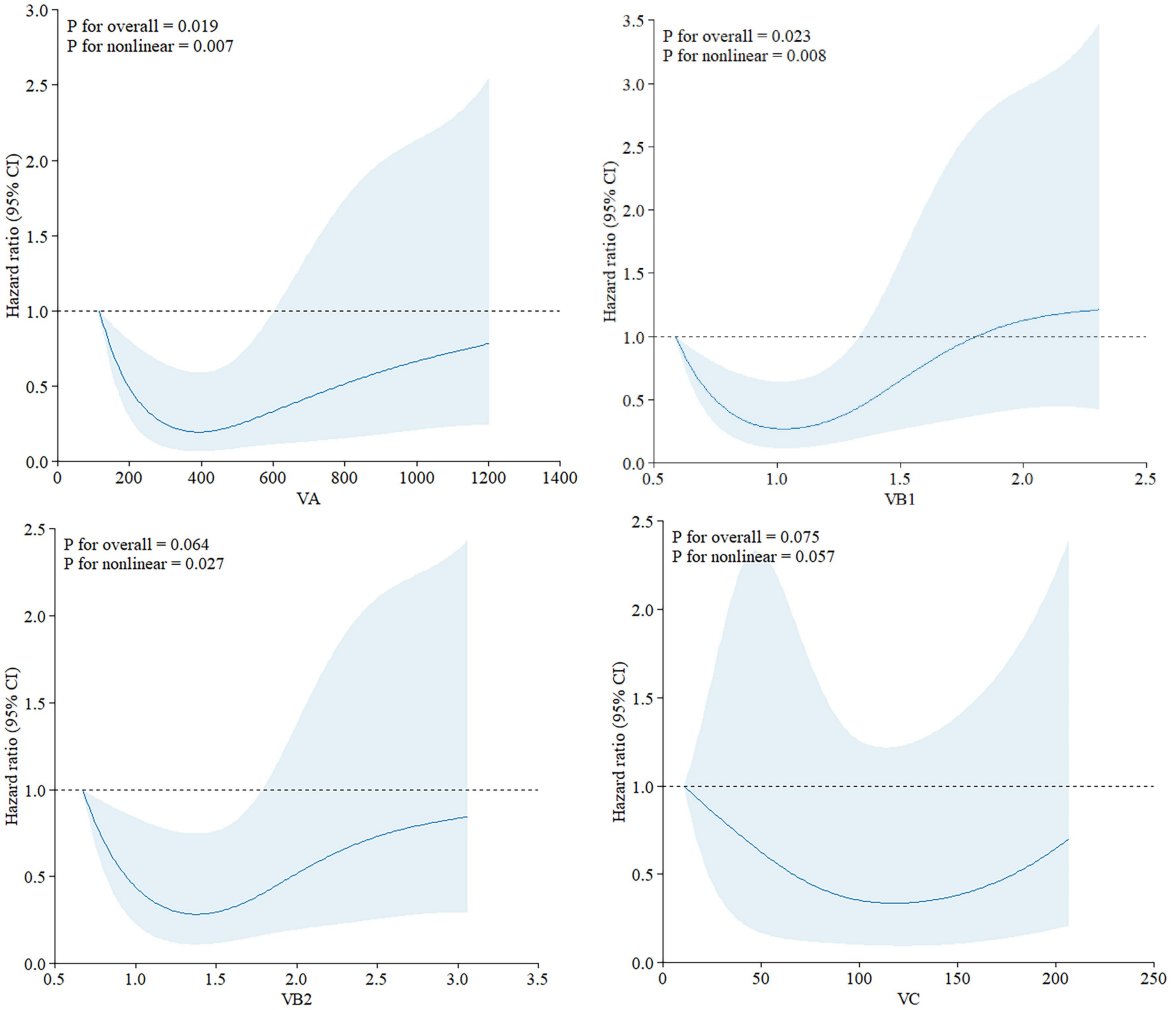
Exposure-effect analysis of dietary vitamins A, B1, B2, and C with all-cause mortality by restricted cubic splines. The exposure effect was fitted by restricted cubic splines with three knots (10, 50, and 90%) after adjusting for age and smoking status. The red lines with the shaded areas embody the estimated HRs of all-cause mortality with 95% CIs.

RCS analyses were conducted to explore the exposure-effect relationships between statistically significant dietary vitamins in Model II and all-cause mortality (Figure 3). Vitamin A (*p*-non-linear = 0.007), vitamin B1 (*p*-non-linear = 0.008), and vitamin B2 (*p*-non-linear = 0.027) exhibited non-linear associations with all-cause mortality in ovarian cancer patients.

### Subgroup analyses

**Figure 4:**
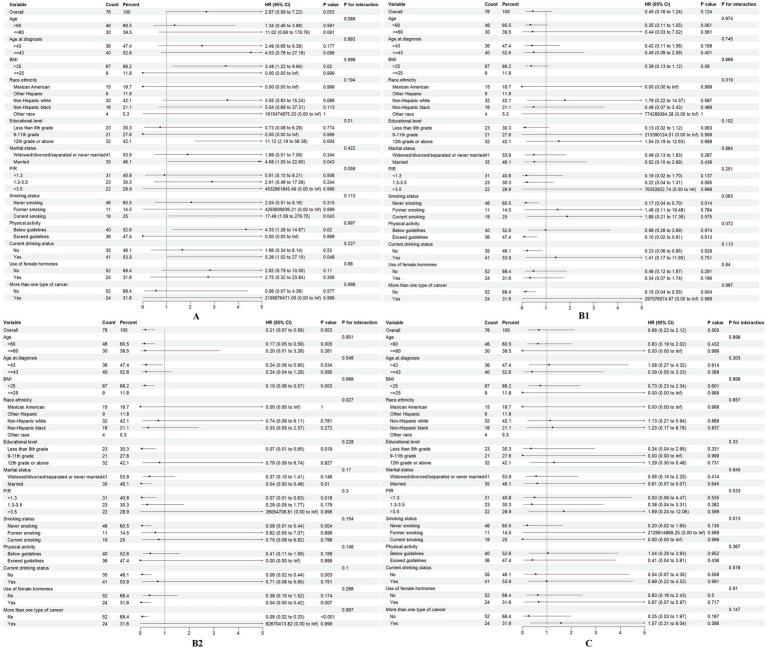
Association of dietary vitamins A, B1, B2, and C (T1 vs. T2-3) with all-cause mortality in different subgroups.

The relationship between dietary vitamins and ovarian cancer survival may vary across different demographic groups. To identify potential confounders, we conducted subgroup analyses for vitamins A, B1, B2, and C, which based on age, BMI, age at diagnosis, race, education level, PIR, smoking status, alcohol consumption, physical activity, marital status, hormone use, and multiple cancer diagnoses (Figure 4).

The summary HR for vitamin A intake was significantly higher in individuals with a 12th-grade education or above (HR = 11.12, 95% CI: 1.61–2.42, *p* = 0.004). In contrast, for those with lower level of education, the HR was not statistically significant, suggesting that vitamin A intake may be more strongly associated with mortality in individuals with higher educational attainment (p for interaction = 0.01).

For vitamins B1 and B2, subgroup analysis revealed differences in racial groups (Vitamin B1: p for interaction = 0.019; Vitamin B2: p for interaction = 0.027), indicating the need for race-adjusted analyses in future studies. Additionally, smoking status emerged as a potential confounder in the relationship between vitamin C intake and ovarian cancer outcomes (*p* for interaction = 0.013).

## Discussion

### Major findings

To our knowledge, this study was the first to comprehensively assess the association between dietary vitamin intake and all-cause mortality in ovarian cancer patients using the NHANES database. Our findings suggested that higher dietary vitamin A intake was linked to an increased mortality risk, while vitamins B1 and B2 was associated with improved survival. The relationship between vitamin C and mortality was more complex, potentially reflecting a threshold effect. These non-linear associations highlighted the nuanced role of dietary vitamins in ovarian cancer survival, which was closely related to their doses.

Previous studies have explored the link between dietary antioxidants and ovarian cancer survival. For example, Sun et al. (6) found that higher vitamin C intake was associated with better survival (HR = 0.43, 95% CI = 0.25–0.75, *p* < 0.05), but they did not assess vitamin A, which we found to have a detrimental effect. Compared to their analysis, our study also included B vitamins, which played a role in tumor prognosis through DNA synthesis and repair mechanisms (13). We also indicate that varying vitamin C doses may have distinct effects on ovarian cancer survival, prompting further investigation into the role of antioxidants in cancer prognosis.

In our subgroup analysis, several demographic factors, such as educational level and race, may confound the association between dietary vitamins and survival outcomes. Future research should focus on stratified studies to better understand how these factors influence vitamin intake and cancer prognosis. Smoking status also emerged as a key factor affecting the role of vitamin C intake to ovarian cancer survival. After adjusting for it, our results still remained robust with consistent findings across models, which again illustrates the robustness of our conclusion.

### Mechanism

The association between high vitamin A intake and increased mortality challenges previous assumptions about its health benefits. While vitamin A is essential for cell differentiation, proliferation, and immune regulation, excessive intake may disrupt these processes, leading to detrimental effects on tumor prognosis. First of all, retinoic acid (RA), the active form of vitamin A, plays a critical role in the signaling pathways of ovarian cancer cells, influencing their differentiation and apoptosis (14). High doses of RA can activate the PI3K/Akt pathway, helping ovarian cancer cells adapt to oxidative stress and promoting their growth and proliferation (15). Besides, high doses of vitamin A can impair normal cell differentiation, promote “dedifferentiation,” and increase cancer risk (16). Moreover, while vitamin A has antioxidant properties (17), excessive antioxidants can enable cancer cells to adapt to oxidative stress, potentially enhancing tumor growth (18). Additionally, long-term high intake of vitamin A may burden the liver, further compromising cancer prognosis (19).

Vitamin B1, a coenzyme in the glycolytic pathway, also plays a key role in the metabolism of ovarian cancer cells. The Warburg effect, where tumor cells rely on glycolysis for energy production even under aerobic conditions, is prominent in ovarian cancer (20). High doses of vitamin B1 can counteract this aerobic glycolytic phenotype, inhibiting tumor growth and inducing apoptosis in cancer cells without affecting normal cells (21). Vitamin B2’s antioxidant effects and its role in DNA repair further support its potential as an anticancer agent (22).

The effects of vitamin C on cancer have long been controversial. Vitamin C is thought to exert multiple anticancer effects. First, vitamin C can enhance cellular and humoral immunity, helping the body build stronger defense mechanisms to fight cancer cells (23). Second, vitamin C promotes collagen synthesis to protect against cancer cell invasion (24). Last but not least, as a powerful antioxidant, vitamin C can neutralize free radicals, reduce oxidative stress, and help reduce cancer cells’ chances of survival (25).

However, vitamin C exhibits both antioxidant and pro-oxidant effects depending on its concentration, with higher levels potentially exerting harmful pro-oxidant effects (26). Our study found that high-dose vitamin C intake (>141.25 mg/day) may exceed recommended levels [a daily VC intake of 75 mg/day for adult women (27)], which could have adverse effects on ovarian cancer prognosis.

### Multicollinearity and model validity

Our assessment of multicollinearity revealed acceptable VIF values, supporting the robustness of our multivariate model. The inclusion of covariates such as age and smoking status enhances the validity of our findings, suggesting that the observed associations are less likely to be confounded by these factors. The crude survival probabilities for each vitamin by Kaplan–Meier curves are consistent with our results, reinforcing the reliability of our analysis.

### Clinical implication

The use of antioxidants in cancer treatment remains controversial (28). As a result, we developed a nomogram predicting ovarian cancer survival based on each dietary vitamin intake, which may be a practical tool for clinicians to make dietary recommendations for each ovarian cancer patient. This approach aligns with the growing emphasis on individualized medicine, offering patients a more tailored and evidence-based care plan.

### Strengths and limitations

This study offered several unique advantages. First, the data used in this study is derived from NHANES, a nationally representative survey, which provides a diverse and representative dataset and enhances the reliability and relevance of our findings. Second, we employed a rigorous statistical approach, identifying adjustment factors through univariate analysis and validating our findings with Kaplan–Meier curves, dose–response analysis, and subgroup analyses. Additionally, we developed a nomogram integrating key variables, providing a practical tool for clinicians to estimate mortality risk based on dietary habits. This enhanced the clinical applicability of our findings, offering healthcare providers a method to deliver personalized dietary recommendations to ovarian cancer patients, potentially improving their prognosis and quality of life. Finally, we conducted a comprehensive investigation into the potential mechanisms linking dietary vitamins to ovarian cancer.

However, several limitations must be acknowledged. First, a major limitation of our study was the small sample size, which may have affected the generalizability and accuracy of the results. For example, as shown in Table 2, the HR for vitamin K in Tertile3 was 0, suggesting a potential progression-free association between specific levels of vitamin K and ovarian cancer. However, due to the limited sample size, we cannot draw definitive conclusions, as the result may be influenced by data sparsity at specific doses, and may not be widely applicable. Second, since data on dietary vitamin intake and ovarian cancer risk factors are self-reported, measurement errors may be present. But given the prospective nature of the data collection, any such errors are likely to be non-differential. Third, due to the lack of data on cancer grade and treatment in the NHANES database, we were unable to adjust for these factors in our analysis. Finally, as an observational study conducted in a single center, it is difficult to draw definitive conclusions about causal relationships.

Future research should aim to capture more detailed information on dietary vitamins and their impact on ovarian cancer prognosis. Additionally, expanding the inclusion of relevant covariates in the analysis could further refine our understanding of factors influencing outcomes in this population.

## Conclusion

In conclusion, our study highlights the complex interplay between dietary vitamin intake and all-cause mortality in ovarian cancer patients. We suggested a potential increased mortality risk associated with higher vitamin A intake, alongside the protective effects of vitamins B1 and B2, underscores the necessity of tailored nutritional interventions in cancer care. However, due to the limited sample size, the statistical power may be insufficient to draw definitive conclusions. The nuanced role of vitamin C with different levels further complicates dietary recommendations. Given these limitations, it is crucial to interpret the results with caution. As we continue to explore the impacts of nutrition on cancer prognosis, future research should aim to include larger, more comprehensive datasets that account for cancer treatment and other relevant covariates, in order to better clarify the role of dietary vitamins in cancer outcomes.

## Data Availability

The original contributions presented in the study are included in the article/Supplementary material, further inquiries can be directed to the corresponding authors.

## Footnotes

^1^ http://wwwn.cdc.gov/nchs/nhanes

^2^ https://www.cdc.gov/nchs/data-linkage/
