# Supplementary material for: Association between dietary vitamin intake and all-cause mortality in ovarian cancer patients: a prospective cohort study
Source: Front Nutr. 2025 May 30;12:1554253. doi: 10.3389/fnut.2025.1554253 (PMC12162896; doi:10.3389/fnut.2025.1554253)

**Supplementary Table 1. Univariate Cox proportional hazard regression model between all covariates and all-cause mortality in ovarian cancer patients, Nation Health and Nutrition Examination Survey 2003–2016**

| **Covariates** | **Meaning of the results** | **HR (95%CI)** | **P** | **P for overall** |
| --- | --- | --- | --- | --- |
| Age | Per 1 SD increase | 1.1 (1-1.1) | 0.002 | - |
| BMI | Per 1 SD increase | 0.99 (0.94-1.1) | 0.83 | - |
| Age at diagnosis | Per 1 SD increase | 1 (0.99-1) | 0.18 | - |
| Race/ethnicity | Mexican American | Ref | - | 0.9 |
|  | Other Hispanic | 0(0-Inf) | Inf |  |
|  | Non-Hispanic white | 1.543(0.438-5.434) | 5.434 |  |
|  | Non-Hispanic black | 2.078(0.536-8.048) | 8.048 |  |
|  | Other race | 1.083(0.113-10.431) | 10.431 |  |
| Educational level | Less than 9th grade | Ref | - | 4 |
|  | 9-11th grade | 0.473(0.145-1.541) | 1.541 |  |
|  | 12th grade or above | 1.008(0.417-2.434) | 2.434 |  |
| PIR | <1.3 | Ref | Ref | 0.7 |
|  | 1.3-3.5 | 1.345(0.533-3.394) | 3.394 |  |
|  | >3.5 | 0.917(0.326-2.581) | 2.581 |  |
| Smoking status | Never smoking | Ref | Ref | 0.08 |
|  | Former smoking | 3.284(1.135-9.498) | 0.028 |  |
|  | Current smoking | 1.159(0.44-3.054) | 0.766 |  |
| Current drinking status | No | Ref | Ref | 0.57 |
|  | Yes | 0.792(0.355-1.769) | 0.57 |  |
| Physical activity | Below guidelines | Ref | Ref | 0.167 |
|  | Exceed guidelines | 0.471(0.163-1.359) | 0.167 |  |
| Marital status | Married | Ref | Ref | 0.75 |
|  | Widowed/divorced/separated or Never married | 0.877(0.392-1.964) | 0.75 |  |
| Use of female hormones | No | Ref | Ref | 0.784 |
|  | Yes | 0.892(0.393-2.023) | 0.784 |  |
| More than one type of cancer | No | Ref | Ref | 0.656 |
|  | Yes | 1.213(0.519-2.837) | 0.656 |  |

Abbreviations: HR: hazard ratio; 95% CI: 95% confidence interval; Ref: reference; Inf: infinity.

**Supplementary Table 2. VIF values of each variable included in fully adjusted multivariate Cox proportional hazard regression model**

| **Variables** | **VIF** |
| --- | --- |
| Vitamin A | 1.97 |
| Vitamin B1 | 2.76 |
| Vitamin B2 | 2.73 |
| Age | 1.41 |
| Smoking status | 1.69 |

**Supplementary Figure 1.** Kaplan–Meier curves of tertiles of dietary vitamin A, B1, B2, and C with all-cause mortality in ovarian cancer patients.


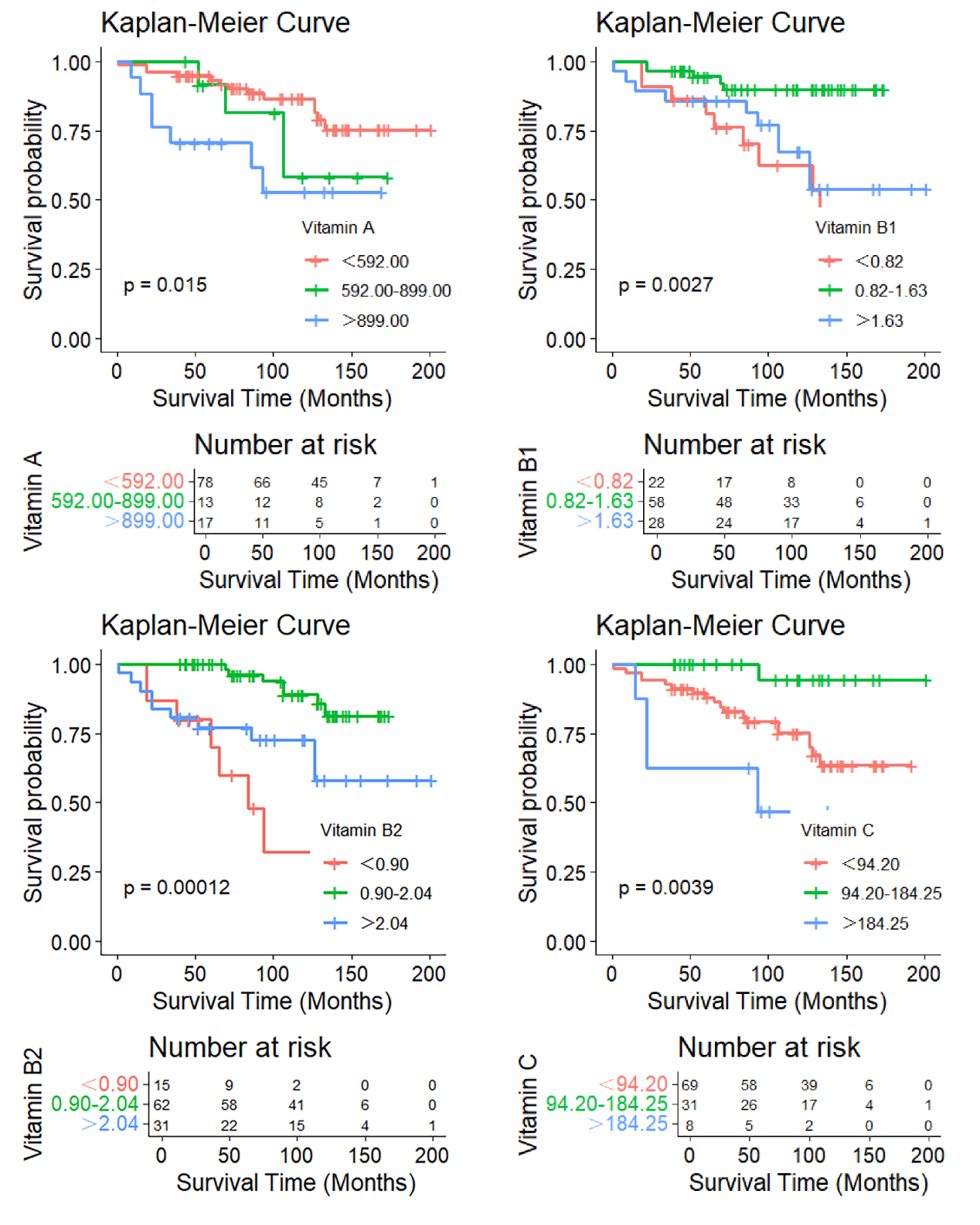

Supplement: Supplementary file 1 [file Table_1.docx]
